# Association of IL-6 rs1800795 (− 174G > C) polymorphism with depression risk: a comprehensive meta-analysis

**DOI:** 10.1038/s41598-026-46667-8

**Published:** 2026-04-01

**Authors:** Xiangliang Wang, Yanwei Cheng, Yongjie Bai, Wenjun Yan, Jinjin Xu, Ruile Shen

**Affiliations:** 1 Department of Neurology, The First Affiliated Hospital, and College of Clinical Medicine of Henan University of Science and Technology, Luoyang, China; 2 Department of Neurology, Henan Provincial People’s Hospital, Zhengzhou, China

**Keywords:** Depression, Interleukin-6, Gene, Polymorphism, Meta-analysis, Diseases, Genetics, Medical research, Risk factors

## Abstract

Depression is a global public health concern with a steadily increasing prevalence. Previous studies examining the association between interleukin-6 (IL-6) gene polymorphisms and depression have produced inconsistent findings. Therefore, we conducted a meta-analysis to clarify the association between IL-6 gene polymorphisms and susceptibility to depression. From eight eligible articles identified, seven studies providing data for the rs1800795 variant were included in the quantitative synthesis. Pooled odds ratios (ORs) with 95% confidence intervals (CIs) were calculated to assess the genetic association under allelic, dominant, recessive, homozygous, and heterozygous genetic models. The meta-analysis revealed no significant association between the IL-6 rs1800795 polymorphism and susceptibility to depression under the allelic, dominant, recessive, homozygous, and heterozygous genetic models (G vs. C, OR = 1.05, 95%CI: 0.83–1.32, *P* = 0.70; GG + CG vs. CC, OR = 1.10, 95%CI: 0.69–1.75, *P* = 0.68; GG vs. CC + CG, OR = 1.11, 95%CI: 0.87–1.43, *P* = 0.40; GG vs. CC, OR = 1.24, 95%CI: 0.79–1.94, *P* = 0.35; CG vs. CC, OR = 1.04, 95%CI: 0.65–1.66, *P* = 0.87). Subgroup analyses stratified by control source and participants’ physical condition also revealed no significant associations under any genetic model. Although no direct association was found, our findings suggest that IL-6 rs1800795 does not confer categorical risk in isolation. Future research should prioritize gene-environment interactions rather than single-locus effects to clarify its role in depression pathogenesis.

**Supplementary Information:**

The online version contains supplementary material available at 10.1038/s41598-026-46667-8.

## Introduction

Depression is a major public health concern and the leading cause of disability worldwide^1^. Its prevalence continues to rise each year^2^. Clinical depression is estimated to affect about 10–15% of the general population over a lifetime^3^. It significantly reduces quality of life and increases the risk of suicide^4^. Research indicates that inflammation and immune responses are associated with the etiology of depression, and this relationship is partly influenced by genetic factors^5^. Depression is thought to arise from an interaction among genetic, environmental, and developmental epigenetic factors^6^. Both genetic and environmental factors play substantial roles in the development of depression^7^.

Depression is a heterogeneous disorder that cannot be fully explained by any single biological or environmental pathway and is thought to arise from a combination of genetic and environmental factors^8^. Contemporary models emphasize converging mechanisms involving stress-response dysregulation, altered neurotransmission, and immune–inflammatory activation^9^. Genetic effects may be context-dependent through gene–environment interplay, which could partly contribute to inconsistent findings across primary studies^10^.

Interleukin-6 (IL-6), encoded by the IL-6 gene^11^, is a key proinflammatory cytokine^12^. It functions as both a cytokine and a myokine within the immune system, influencing multiple autoimmune diseases^4^. Evidence suggests that cytokine-mediated signaling pathways between the immune system and the brain contribute to the pathogenesis of depression^13^. The IL-6 gene consists of four introns and five exons located on chromosome 7p15-p21^4^. Moreover, the promoter region of the IL-6 gene contains three single-nucleotide polymorphisms (rs1800795, rs1800796, and rs1800797) thought to regulate IL-6 expression^14,15^. Previous studies have identified the IL-6 gene as a candidate region associated with major depressive disorder (MDD), and its genetic mechanisms underlying depressive symptoms have been further explored^16^.

The association between IL-6 gene polymorphisms and depression risk has been extensively studied, yet findings remain inconclusive. Some studies suggest that the IL-6 rs1800795 polymorphism may serve as a molecular biomarker for MDD^4^, and is associated with an increased risk of depression^17^. The IL-6 rs1800796 promoter variant directly influences IL-6 transcription and expression^4^. However, Hong et al. reported that the examined IL-6 polymorphism does not influence MDD susceptibility^18^. Therefore, the primary objective of this meta-analysis was to estimate the pooled association between the IL-6 promoter variant rs1800795 (− 174G > C) and depression susceptibility. We additionally performed prespecified subgroup analyses by source of controls (hospital-based vs. population-based) and participants’ physical condition (comorbid vs. non-comorbid) to explore the potential influence of gene-environment interactions on depression risk.

## Materials and methods

This meta-analysis was performed according to the Preferred Reporting Items for Systematic Reviews and Meta-Analyses guidelines^19^ and was registered at the International Prospective Register of Systematic Reviews (CRD42023456681).

Search strategy. We conducted a systematic literature search in PubMed, Embase, Ovid, the Cochrane Library (including the Cochrane Central Register of Controlled Trials), and Web of Science to identify eligible articles from inception to September 21, 2025. The search employed a combination of MeSH terms and free-text keywords including: (“Depression” OR “Depressive Disorder”) AND (“Interleukin-6” OR “IL-6”) AND (“Polymorphism” OR “Genetic Variation” OR “SNP”). To ensure comprehensiveness, the reference lists of retrieved articles and previous meta-analyses were manually screened. Detailed search strings for each database are provided in Supplementary appendix 1.

Inclusion criteria and exclusion criteria. Studies were included if they met the following criteria: (1) investigated the association between IL-6 polymorphisms and depression; (2) used a case-control design; (3) provided sufficient data on cases and controls to calculate odds ratios (ORs) with 95% confidence intervals (CIs); (4) reported genotype distributions in control groups consistent with Hardy-Weinberg equilibrium (HWE); and (5) in cases of duplicate or overlapping data, the most recent or most comprehensive publication was selected. We anticipated diagnostic heterogeneity across primary studies (e.g., different diagnostic criteria or symptom scales used to define depression). Therefore, we extracted the ascertainment method for depression in each study and considered this variability as a prespecified methodological limitation when interpreting pooled estimates. The exclusion criteria were: (1) duplicate publications; (2) incomplete data; (3) the genotype distribution in the control group deviated from HWE; and (4) case reports, reviews, letters, or meta-analyses. Titles and abstracts were initially screened, and studies that clearly failed to meet the inclusion criteria were excluded. Studies that provisionally met the inclusion criteria underwent full-text review for further eligibility assessment. Two reviewers independently assessed the articles and resolved any disagreements through discussion. Only the most relevant articles were included in the final analysis.

Data extraction. Two investigators independently extracted data from each included publication. Discrepancies were resolved through discussion between the two reviewers, and if no consensus was reached, a decision was made by all reviewers. Extracted data included: first author’s name, year of publication, country, source of controls, ethnicity of the study population, genotype counts in cases and controls, and HWE status in control groups. Crucially, to address the pre-identified methodological limitation of between-study diagnostic heterogeneity, we systematically documented the specific methods used to ascertain depression (diagnostic criteria and/or validated rating scales) and the participants’ physical comorbidities. If any relevant data were missing, study authors were contacted to obtain the information.

Quality assessment. Study quality was independently assessed by two revi ewers using the Newcastle-Ottawa Scale (NOS). Disagreements were resolved through discussion or adjudicated by a third reviewer to ensure consistency.

Statistical analysis. The association between IL-6 and depression risk was assessed by calculating pooled ORs with 95% CIs. HWE in control groups was tested using the χ² test, with *P* < 0.05 indicating statistical significance. The allelic, dominant, recessive, homozygous and heterozygous genetic models were used to calculate the combined ORs. Heterogeneity was assessed using the Q-test and *I²* statistic. Heterogeneity was considered significant if *P* < 0.1 or *I²* > 50%. Given the potential between-study heterogeneity, a random-effects model was applied in the meta-analysis. A continuity correction of 0.5 was applied to studies with zero events^20^. Prespecified subgroup analyses (as outlined in the study protocol/PROSPERO registration, CRD42023456681) were conducted to explore potential sources of heterogeneity. Subgroups were defined a priori by (i) source of controls (hospital-based vs. population-based) and (ii) physical condition of participants (comorbid vs. non-comorbid). Studies were categorized as “comorbid” when participants had a specified medical condition (e.g., cardiovascular disease, diabetes, hemodialysis, chronic hepatitis C) and as “non-comorbid” otherwise. Sensitivity analysis was performed by sequentially removing one study at a time to evaluate result stability. Publication bias was assessed using funnel plots and Egger’s test. All statistical analyses were performed using STATA version 17.0 (StataCorp, College Station, TX, USA). A *P*-value < 0.05 was considered statistically significant.

## Results

**Fig. 1:**
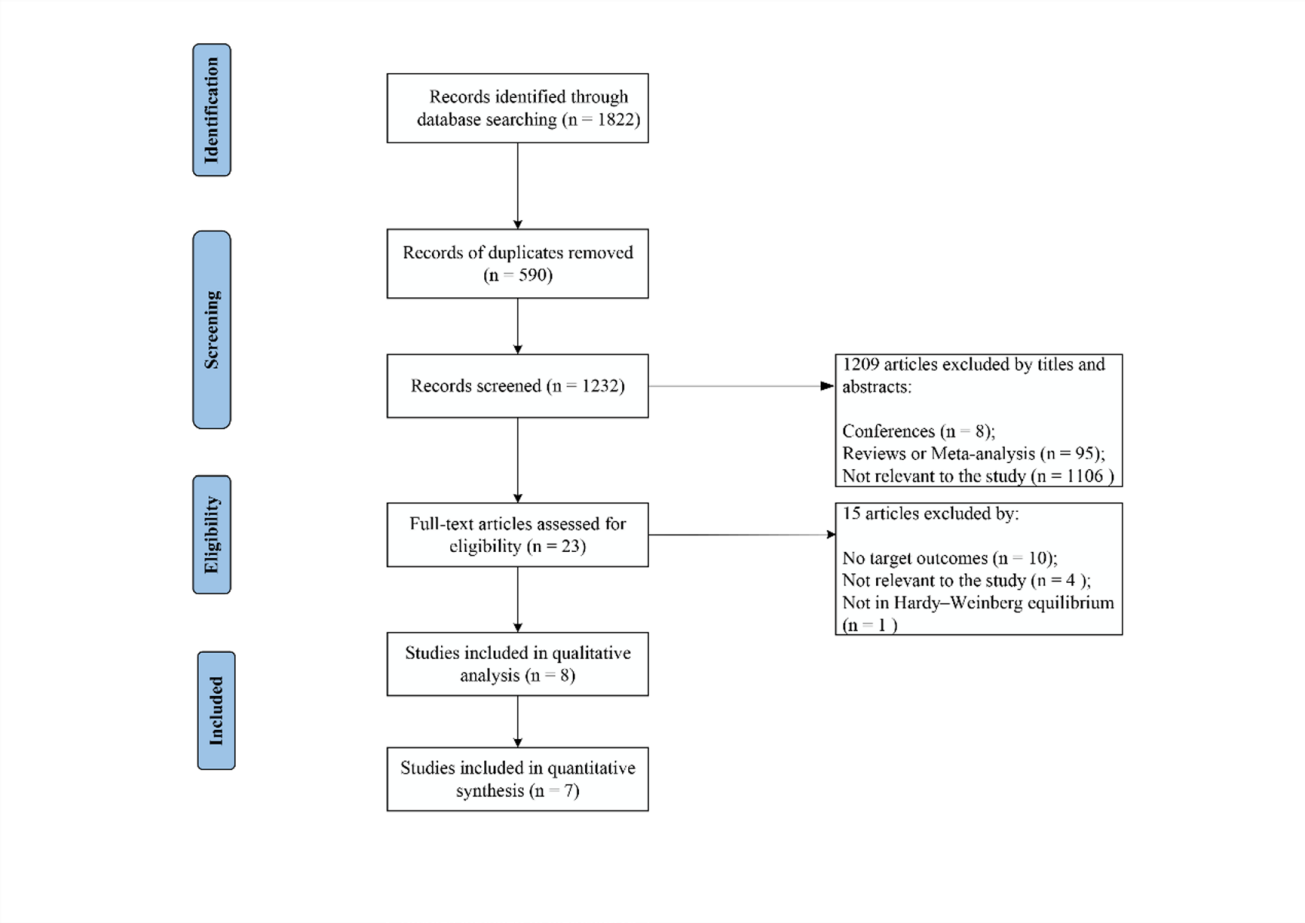
PRISMA flowchart for the study inclusion.

Characteristics of included studies. A total of 1,822 articles were initially identified through database searching. After removing 590 duplicates, 1,209 articles were excluded based on their titles and abstracts. The remaining 23 articles underwent full-text assessment. One study was excluded because the genotype distribution in the control group deviated from HWE^21^. Ultimately, eight eligible case-control studies were included in the qualitative systematic review^16,18,22–27^. However, as our quantitative synthesis focused on the rs1800795 (− 174G > C) variant, one study that investigated other IL-6 SNPs but lacked data for rs1800795 was excluded from the meta-analysis^16,22–27^. A flowchart detailing the study selection process is shown in (Fig. 1).

Ultimately, eight case-control studies comprising 3,204 individuals (1,668 cases and 1,536 controls) met the inclusion criteria and were included in the meta-analysis. Seven studies examined the association between IL-6 rs1800795 and depression susceptibility; two investigated rs1800796; and one each investigated rs1800797, rs2069837, and rs1524107. The characteristics of the included studies are summarized in Table 1. Among the included studies, two were conducted in China, two in Poland, two in Russia, one in Bulgaria, and one in Jordan. Regarding ethnicity, six studies were conducted in Caucasian populations and two in Chinese populations. Based on control source, four studies used population-based samples and four used hospital-based samples. With respect to physical condition, five studies included non-comorbid populations, and three included comorbid populations. Genotype and allele distributions of the IL-6 gene in cases and controls are presented in Table 1.

**Table 1:** Main characteristics of studies included in the meta-analysis.

Author		Country	Ethnicity	Cases	Controls	Diagnosis of depression	Associated diseases	Cases					Controls					HWE *P* value	NOS	SOC
	Genotype = rs1800795							CC	CG	GG	C	G	CC	CG	GG	C	G			
Adler et al., 2016^19^		Poland	Caucasian	91	206	GDS	Normal	26	41	24	93	89	43	106	57	192	220	0.627	8	HB
Alshogran et al., 2019^20^		Jordan	Caucasian	160	136	HADS	Hemodialysis	13	74	73	100	220	21	59	56	101	171	0.409	8	HB
Frydecka et al., 2016^21^		Poland	Caucasian	24	38	DSM-Ⅳ	Chronic Hepatitis	5	12	7	22	26	5	21	12	31	45	0.374	8	HB
Gafarov et al., 2022^22^		Russia	Caucasian	206	149	MONICA-MOPSY	Normal	36	90	80	162	250	36	70	43	142	156	0.477	9	PB
Golimbet et al., 2018^23^		Russia	Caucasian	78	91	HAM-D-21	Ischemic Heart Disease	6	43	29	55	101	14	46	31	74	108	0.650	8	HB
Mihailova et al., 2016^24^		Bulgaria	Caucasian	80	52	HAM-D	Normal	18	28	34	64	96	7	25	20	39	65	0.853	9	PB
Zhang et al., 2016^13^		China	Chinese	772	759	DSM-Ⅳ	Normal	0	16	756	16	1528	0	7	752	7	1511	0.879	9	PB
	Genotype = rs1800796							GG	GC	CC	G	C	GG	GC	CC	G	C			
Hong et al., 2005^15^		China	Chinese	257	105	DSM-Ⅳ	Normal	10	86	161	106	408	4	38	63	46	164	0.554	9	PB
Zhang et al., 2016^13^		China	Chinese	772	759	DSM-Ⅳ	Normal	56	346	370	458	1086	64	320	375	448	1070	0.713	9	PB
	Genotype = rs1800797							AA	AG	GG	A	G	AA	AG	GG	A	G			
Zhang et al., 2016^13^		China	Chinese	772	759	DSM-Ⅳ	Normal	0	19	753	19	1525	0	4	755	4	1514	0.942	9	PB
	Genotype = rs2069837							AA	AG	GG	A	G	AA	AG	GG	A	G			
Zhang et al., 2016^13^		China	Chinese	772	759	DSM-Ⅳ	Normal	481	269	22	1231	313	483	251	25	1217	301	0.269	9	PB
	Genotype = rs1524107							CC	CT	TT	C	T	CC	CT	TT	C	T			
Zhang et al., 2016^13^		China	Chinese	772	759	DSM-Ⅳ	Normal	58	354	360	470	1074	64	334	361	462	1056	0.280	9	PB

HWE, Hardy-Weinberg equilibrium; NOS, the Newcastle-ottawa Scale; SOC, source of control; GDS, Geriatric Depression Scale; HADS, Hospital Anxiety and Depression Scale; DSM-Ⅳ, Diagnostic and Statistical Manual of Mental Disorders, Fourth Edition; MOPSY, MONICA (MONItoring of trends and determinants in Cardiovascular disease) Optional Psychosocial Substudy; HAM-D-21, the 21-point Hamilton Depression Scale; HAM-D, Hamilton Depression Rating Scale; HB, hospital-based; PB, population-based.

**Fig. 2:**
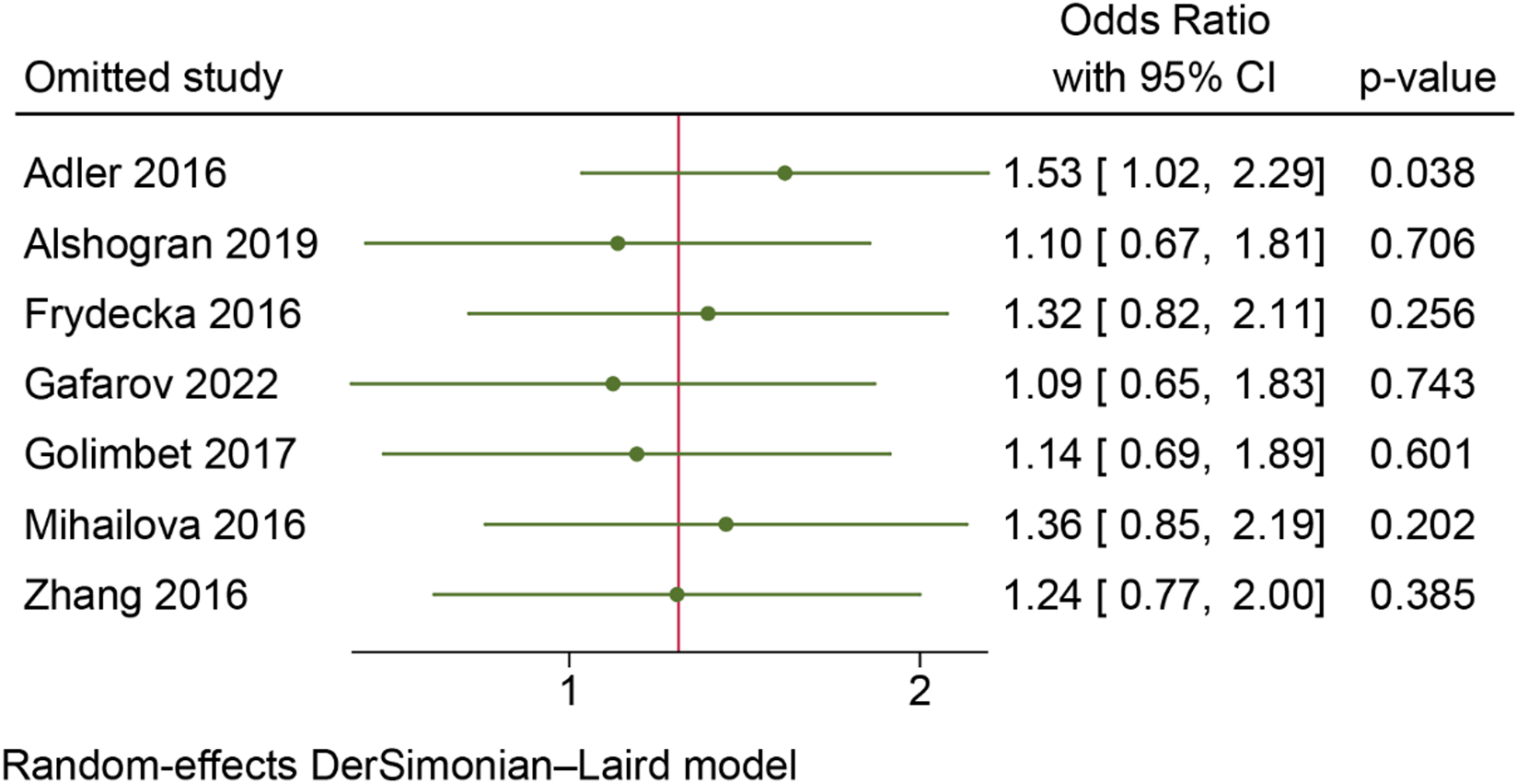
Sensitivity analysis under homozygous model (GG vs. CC).

Quality assessment, sensitivity analysis and publication bias. The NOS was used to assess the quality of 8 studies, with results presented in Table 1. All included studies achieved satisfactory NOS scores ranging from 7 to 9, indicating good methodological quality. Sensitivity analysis was conducted to assess the robustness of the results by sequentially removing individual studies. The pooled ORs remained largely unchanged in the sensitivity analysis, except under the homozygous model (GG vs. CC) in the overall population (Fig. 2), indicating that the findings of this meta-analysis were generally robust. Funnel plot shapes across all genetic models showed no clear evidence of asymmetry (Supplementary appendix 1). Similarly, Egger’s test revealed no significant publication bias in any genetic model for the overall population. (Table 2).

**Table 2:** Meta-analysis of IL-6 gene polymorphisms and susceptibility to depression.

Analysis model	Study groups	Studies	ORs (95% CIs)	*P*_OR_	*P*_Egger_	Heterogeneity	
						*P*	I^2^ (%)
G vs. C	Total analysis	7	1.05 (0.83–1.32)	0.70	0.074	0.08	47.64
	HB	4	1.06 (0.83–1.37)			0.24	29.16
	PB	3	0.93 (0.53–1.64)			0.03	71.45
	Comorbidity	3	1.22 (0.94–1.57)			0.51	0
	Non-comorbidity	4	0.93 (0.62–1.37)			0.03	67.34
GG + CG vs. CC	Total analysis	7	1.10 (0.69–1.75)	0.68	0.827	0.06	49.98
	HB	4	1.15 (0.57–2.34)			0.04	64.87
	PB	3	1.03 (0.48–2.21)			0.19	40.23
	Comorbidity	3	1.57 (0.80–3.10)			0.23	32.93
	Non-comorbidity	4	0.88 (0.50–1.56)			0.11	49.46
GG vs. CC + CG	Total analysis	7	1.11 (0.87–1.43)	0.40	0.088	0.32	13.89
	HB	4	1.08 (0.80–1.46)			0.90	0
	PB	3	1.02 (0.51–2.01)			0.05	67.60
	Comorbidity	3	1.15 (0.81–1.63)			0.89	0
	Non-comorbidity	4	1.02 (0.64–1.63)			0.08	55.40
GG vs. CC	Total analysis	7	1.24 (0.79–1.94)	0.35	0.608	0.15	36.43
	HB	4	1.21 (0.62–2.38)			0.10	52.45
	PB	3	1.28 (0.61–2.67)			0.24	29.41
	Comorbidity	3	1.69 (0.91–3.16)			0.32	12.31
	Non-comorbidity	4	1.02 (0.55–1.93)			0.13	46.29
CG vs. CC	Total analysis	7	1.04 (0.65–1.66)	0.87	0.993	0.08	46.17
	HB	4	1.14 (0.56–2.32)			0.05	61.58
	PB	3	0.90 (0.40–2.02)			0.19	39.71
	Comorbidity	3	1.58 (0.81–3.08)			0.26	24.93
	Non-comorbidity	4	0.81 (0.47–1.38)			0.19	36.30

ORs, odd ratios; CIs, confidence intervals.

Meta-analysis results. Our systematic search initially identified eight eligible studies investigating various IL-6 polymorphisms. However, as only seven of these studies provided data for the rs1800795 (− 174G > C) variant, our quantitative synthesis focused exclusively on this polymorphism. The remaining four SNPs (rs1800796, rs1800797, rs2069837, and rs1524107) were excluded from the meta-analysis due to an insufficient number of studies for meaningful pooling. Accordingly, seven case-control studies involving 1,411 cases and 1,431 controls assessed the association between IL-6 rs1800795 polymorphism and depression susceptibility^16,22–27^. No significant association between the IL-6 rs1800795 polymorphism and depression susceptibility was found in this meta-analysis under allelic, dominant, recessive, homozygous, and heterozygous genetic models (G vs. C, OR = 1.05, 95%CI: 0.83–1.32, *P* = 0.70; GG + CG vs. CC, OR = 1.10, 95%CI: 0.69–1.75, *P* = 0.68; GG vs. CC + CG, OR = 1.11, 95%CI: 0.87–1.43, *P* = 0.40; GG vs. CC, OR = 1.24, 95%CI: 0.79–1.94, *P* = 0.35; CG vs. CC, OR = 1.04, 95%CI: 0.65–1.66, *P* = 0.87) (Supplementary appendix 1). Prespecified subgroup analyses were performed by control source (hospital-based vs. population-based) and participants’ physical condition (comorbid vs. non-comorbid), and similarly showed no significant association between the IL-6 rs1800795 polymorphism and depression (Table 2).

## Discussion

In this meta-analysis we found that the IL-6 rs1800795 (− 174G > C) polymorphism was not significantly associated with an increased risk of depression. This conclusion differs from some individual studies^23,24,28^, as carriage of the G allele in the IL-6 rs1800795 gene doesn’t increase the susceptibility to depression of the individuals with chronic diseases. Results were broadly robust to sensitivity analyses, although the homozygous comparison showed some instability. Stratifying by whether control groups had psychiatric comorbidity did not materially change the estimates, suggesting that control composition alone is unlikely to account for the null association observed here.

Our findings are consistent with a wider literature indicating that single common IL6 variants may not exert large main effects on categorical depression risk, even though IL‑6 biology remains relevant to depressive states. Elevated IL‑6 concentrations have been observed in depressive episodes, including in cerebrospinal fluid and population-based cohorts, reinforcing biological plausibility for neurobehavioral effects^29,30^. Imaging genetics work has further linked IL6 variation to brain structure in healthy adults, suggesting pleiotropic, context‑dependent actions of IL‑6 on the brain^31^. Reports of altered Hypothalamic-Pituitary-Adrenal axis dynamics in MDD also implicate IL-6 signaling in clinically relevant neuroendocrine changes^32^.

Several prior meta-analyses have synthesized the relationship between inflammation and depression, often focusing on circulating IL-6 levels rather than IL6 genetic variants. For example, longitudinal evidence suggests that elevated IL-6/CRP has been associated with subsequent depressive symptoms in some populations, supporting biological relevance of inflammatory signaling to depressive phenotypes^33^. However, biomarker-level associations do not necessarily imply that a single common IL6 promoter variant confers a detectable main effect on categorical depression risk. Therefore, our null findings for rs1800795 can be reconciled with the broader inflammatory literature, and may reflect small effect sizes, phenotypic/diagnostic heterogeneity, and context dependence that require larger harmonized genetic datasets to resolve.

The lack of a direct association observed in our study likely reflects the multifaceted regulatory mechanisms of IL-6. As a pleiotropic mediator of inflammation, the impact of IL-6 variants on the central nervous system is context-dependent rather than linear. Its neurobehavioral effects may be masked or moderated by environmental variables—such as psychosocial stress, social support systems, and somatic comorbidities—as well as epistatic interactions with other genetic loci. Consistent with this view, human studies show that rs1800795 can interact with stress exposure and pain burden to shape depressive phenotypes, with signals often captured under additive or recessive models^17^. Prospective data in youth likewise suggest that interpersonal stress moderates associations between IL-6 pathway variation and depressive symptoms, in line with a stress-inflammation coupling framework^34^. Taken together, these findings support a network view in which IL-6-related genetic variation calibrates symptom profiles under specific exposures rather than conferring categorical disease risk by itself.

Mechanistically relevant clinical correlates help contextualize heterogeneity across primary studies. Central IL-6 elevations and Hypothalamic-Pituitary-Adrenal axis alterations provide convergent biological context, while morphometric associations in hippocampal regions align with potential neuroplastic effects^29,31,32^. These considerations may help explain heterogeneous single‑study results when phenotypes, exposures, and clinical states differ.

From a clinical standpoint, the absence of a reliable main‑effect association for rs1800795 argues against routine genotyping of this single SNP as a risk marker for MDD. Nevertheless, IL‑6 remains clinically relevant: higher plasma IL‑6 has been associated with more refractory depression in antidepressant‑treated cohorts, and − 174G > C has been reported to relate to antidepressant outcomes in some samples^35,36^. These observations suggest that integrating protein measures, exposure profiles (stress/pain/medical comorbidity), and multi‑variant genetic information m ay offer more prognostic value than any single variant alone. In immune‑therapy contexts, cytokine‑related neuropsychiatric symptoms underscore the salience of inflammatory pathways for somatic‑dominant depressive features^37^.

Several limitations of this meta-analysis should be considered when interpreting the findings. Firstly, only published articles were included, which may have introduced publication bias. Secondly, gene-environment interactions were not comprehensively assessed in this meta-analysis. Thirdly, diagnostic criteria for depression varied across studies, which could have affected the results. Such diagnostic heterogeneity (differences in diagnostic criteria and/or symptom-scale thresholds) may have contributed to between-study variability and attenuated detectable main genetic effects. Finally, the overall sample size remained relatively small. Therefore, additional well-designed case-control studies with larger sample sizes and detailed participant characteristics are warranted.

## Conclusions

This meta-analysis indicates that the IL-6 rs1800795 polymorphism is not a standalone risk factor for developing depression. However, these negative findings offer a critical perspective for precision medicine: the etiology of depression likely involves complex gene-environment interplay rather than single-locus effects. Future research should therefore prioritize specific subpopulations and integrate environmental exposures to elucidate the latent role of IL-6 in the pathogenesis of depression.

## Supplementary Information

Below is the link to the electronic supplementary material.

Supplementary Material 1

## Data Availability

Data is provided within the manuscript or supplementary information files.

## Abbreviations

IL-6: Interleukin-6
ORs: Odds ratios
CIs: Confidence intervals
